# Absent Thumb and Radius in a Neonate With Tracheo-Esophageal Fistula and Ventricular Septal Defect: VACTERL Association

**DOI:** 10.7759/cureus.51058

**Published:** 2023-12-25

**Authors:** Anshul Sood, Gaurav V Mishra, Shreya Khandelwal, Manasa Suryadevara, Nishtha Manuja

**Affiliations:** 1 Radiodiagnosis, Jawaharlal Nehru Medical College, Datta Meghe Institute of Higher Education and Research, Wardha, IND; 2 Radiodiagnosis, Datta Meghe Institution of Higher Education and Research, Wardha, IND; 3 Radiodiagnosis, Datta Meghe Institute of Higher Education and Research, Wardha, IND; 4 Internal Medicine, Jawaharlal Nehru Medical College, Datta Meghe Institute of Higher Education and Research, Wardha, IND

**Keywords:** vacterl-h, vater, radial ray, neonate, vacterl

## Abstract

A complex of anomalies involving the vertebral column and spinal canal (V), anal atresia(A), congenital lesions of the heart (C), defects involving the trachea esophageal complex (TE), renal system, and urinary tract (R), and limb lesions (L) is known as VACTERL complex. VACTERL is an umbrella term for patients with abnormalities involving three or more of the systems mentioned above. It can be potentially life-threatening and should be promptly recognized and managed. Thorough investigations are required to prevent long-term sequelae and to improve morbidity. We present a case of a neonate born to a mother with twin gestation at 38 weeks of gestation with antenatally diagnosed severe polyhydramnios and a single umbilical artery and vein. This manuscript discusses the imaging findings of the congenital abnormalities involving the cardiac and skeletal system with tracheoesophageal fistula in our patient.

## Introduction

VACTERL association is an acronym for a condition characterized by sporadic, non-random association of specific congenital disabilities of multiple organ systems [1]. The incidence of VACTERL association is 1 in 10,000 to 1 in 40,000 live births [2]. VACTERL complex stands for vertebral column and spinal canal anomalies (V), gastrointestinal tract anomalies (A), congenital lesions of the heart (C), defects involving trachea-esophageal involvement (TE), anomalies of the renal system and urinary tract (R), and limb lesions (L) [3]. For diagnosing the newborn with VACTERL association, abnormalities involving at least three of the abovementioned systems are required. Sometimes, this complex is associated with hydrocephalus, and this rephrases the term as VACTERL-H [3].

## Case presentation

A 1.7 kg male baby with an APGAR score of 10 at one and five minutes, born to a G2P1L1 mother with twin gestation with 38 weeks of gestation, was delivered by cesarean section. The baby cried immediately after birth.

**Figure 1:**
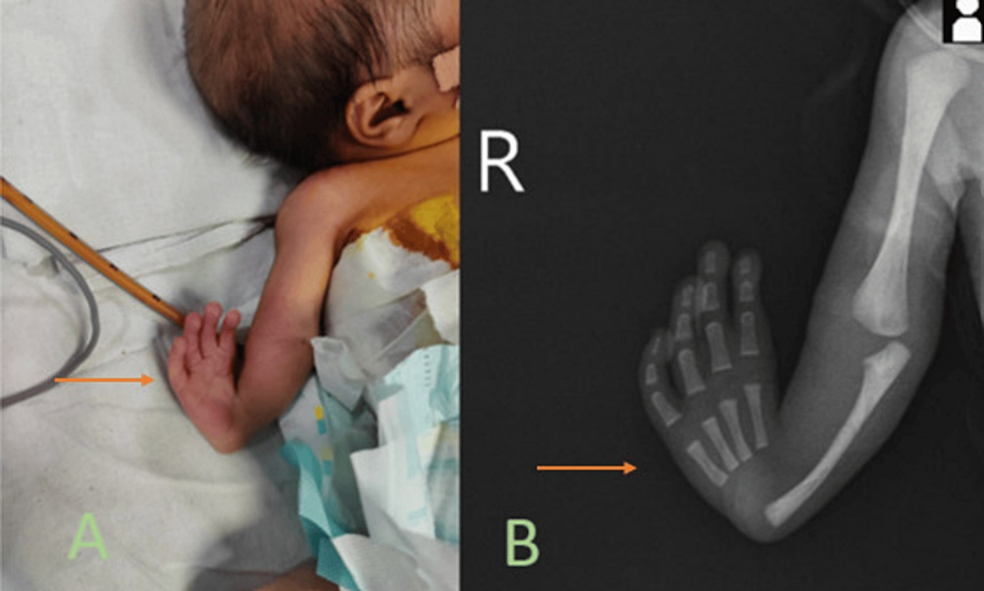
Clicked Image (A) and Antero-posterior X-ray of right arm showing complete absence of the radius and thumb of the right hand suggestive of radial ray type 4 with radial deviation of the fingers (orange arrows).

On general examination of the patient at birth, there were normal nasal, oral, and anal openings. The right thumb and radial deviation of the upper limb was absent, as shown in Figure 1A. On the x-ray of the forearm, the radius was absent with an absent thumb, suggesting it to be radial ray type 4, as shown in Figure 1B.

Reports of previously done antenatal scans revealed a single umbilical artery and a single umbilical vein with severe polyhydramnios. Doppler parameters were within normal limits.

**Figure 2:**
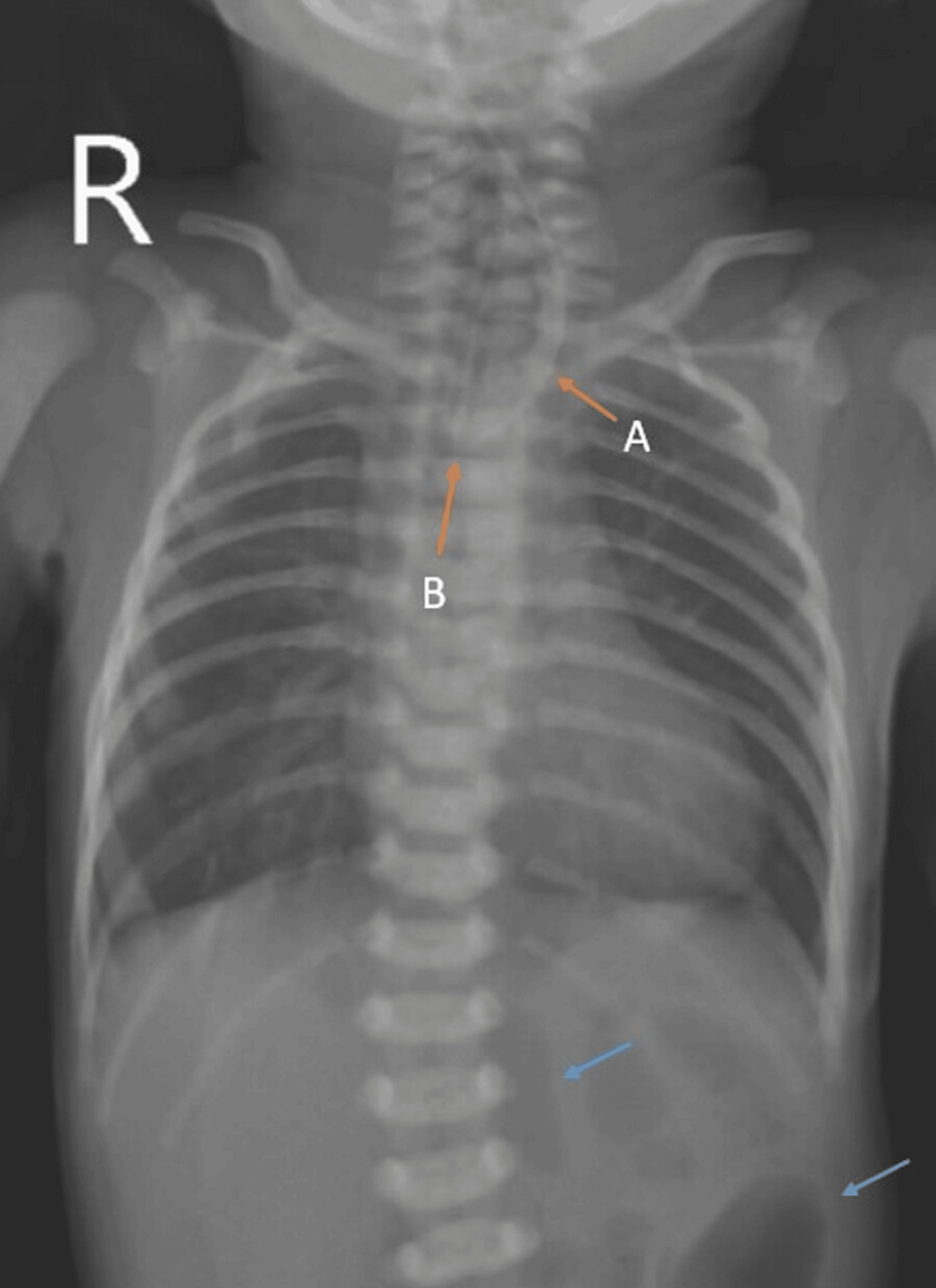
Chest X-Ray of the patient showing (A) nasogastric tube in the esophagus, and (B) coiling of the nasogastric tube within the esophagus (orange arrows) with presence of gas in the bowel (blue arrows) suggestive of proximal esophageal atresia with distal tracheoesophageal fistula.

The baby was not accepting breastfeeds provided by the mother and was vomiting any intake of the feed, which was associated with coughing and cyanosis. The pediatrician advised inserting a nasogastric tube to check the patency of the food pathway. On inserting the nasogastric tube on post op day (POD) 1, resistance was felt, and coiling of the nasogastric tube was noted on the chest x-ray with the presence of gas in the bowel loops, suggesting it to be a proximal esophageal atresia with distal tracheo-esophageal fistula most likely representing type-C, as shown in Figure 2.

**Figure 3:**
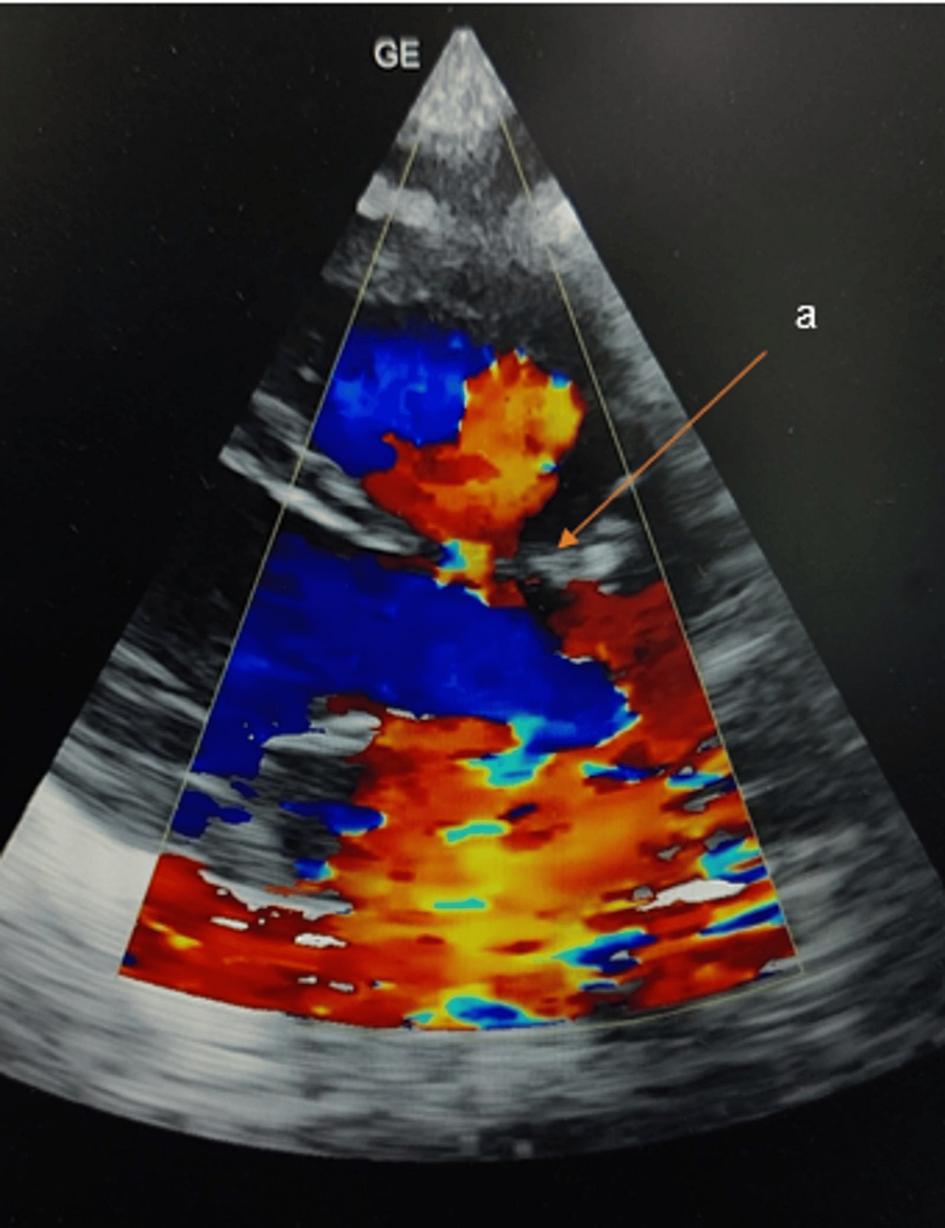
2D echo of the patient showing peri-membranous ventricular septal defect with bi-directional shunt (orange arrow)

To rule out suspicion of VACTERL complex association, sonography of the whole abdomen and spine, neurosonogram, and 2D echo were advised. The 2D echo revealed a moderate-sized peri-membranous ventricular septal defect of approximately 9mm with a bidirectional shunt, as shown in Figure 3. A cardiology call was made for the same, who advised an increase in calorie intake and the use of furosemide for six months. A follow-up was advised after three months of age or in case of any emergency.

Sonography of the abdomen and spine and neuro-sonogram revealed no obvious abnormalities. Spine X-ray was unremarkable. Anal atresia was not present. The patient underwent right postero-lateral thoracotomy with ligation of TEF type-C with esophagostomy with gastrostomy with long gap esophageal atresia with ICD insertion. Post-op, the baby was intubated and shifted to the NICU. The baby's well-tolerated gastrostomy feeds.

An orthopedic opinion was taken for the limb defects. The baby's mother was advised to do passive stretching exercises of the hand towards the ulnar side and follow up after one month. The baby was advised to be discharged from the hospital with a review after one month for the deformity in the right hand and after one and a half months for a change of the gastrostomy tube. The patient was discharged on the POD 8, and the weight was 1.840 kg at discharge. The second baby was completely normal and had no significant abnormalities.

## Discussion

In 1973, to establish the association specificity of the group of congenital malformations, including vertebral defects, anal atresia, tracheo-esophageal fistula, and renal and radial abnormalities, the term VATER was introduced. Later, the term was expanded to be known as VACTERL, which additionally includes cardiac defects and any limb anomalies [4]. People are diagnosed with VACTERL when there is an association of three or more anomalies [5].

Vertebral anomalies are seen in approximately 70 percent of the patients with VACTERL association [6]. These can be classified as {1} failure of formation, which may include butterfly vertebra, hemivertebra; {2} failure of segmentation - block vertebra, fused vertebra; and {3} mixed deformity, which includes a combination of both [7,8]. These deformities may lead to abnormal curvature of the spine and cause severe back pain. During the prenatal period, sonography is the safe and inexpensive modality of choice for diagnosing vertebral and spinal anomalies. Postnatally, when the condition does not require an emergency intervention, a detailed MRI of the spine and brain should be performed [9].

Anal atresia or narrowing of the anal canal is very common and is seen in approximately 60 percent of patients with VACTERL complex association [5]. For imaging of pelvic structures, internal fistulas, and distal rectal pouch, ultrasonography (US) is an excellent diagnostic modality. It is widely being used as a screening method for associated genito-urinary and spinal anomalies [10]. An early surgical intervention is required for this anomaly.

Cardiac defects account for approximately 75 percent of the patients with VACTERL association. Most commonly, these include tetralogy of Fallot, atrial septal defect, and ventricular septal defects. Less commonly, truncus arteriosus and transposition of the great arteries are noted [6]. The primary modality of choice for diagnosing congenital heart defects is echocardiography, which has excellent diagnostic accuracy [11].

The ventricular septum comprises five components: muscular, membranous, infundibular, inlet, and atrioventricular. Ventricular septal defects are classified into four subtypes based on the failure of fusion of any of these components as {1} type 1 - conoventricular septal defect - defect in the ventricular septum where the septum should meet just below the aortic and pulmonary valves; {2} type 2 - peri-membranous ventricular septal defect- defect in the upper section of the ventricular septum; {3} type 3 - Inlet ventricular septal defect - defect in the septum near to the mitral or tricuspid valve; and {4} type 4 - muscular ventricular septal defect - it is the most common type and involves a defect in the muscular part of the ventricular septum [12]. In our patient, there was the presence of a peri-membranous type of ventricular septal defect.

Esophageal atresia with tracheo-esophageal fistula is seen in about 70 percent of the patients with VACTERL association [6]. One of the earliest signs of TEF is polyhydramnios, or absent gastric bubble noted prenatally. Postnatal signs may include choking or swallowing or inability to pass the nasogastric tube immediately postnatally [2]. Chest X-Ray is the primary diagnostic modality that shows the presence of a curled nasogastric tube in patients with unsuccessful passage of a nasogastric tube [13]. An early surgical approach is a must to treat the fistula and obtain a proper feeding route.

Tracheo-esophageal fistula / esophageal atresia can be classified as {1} type A - atresia of the esophagus; {2} type B - distal atresia of the oesophagus with proximal fistulous connection with trachea; {3} type C - proximal atresia of the oesophagus with distal fistulous connection with the trachea; {4} type D - intervening atresia with double fistulous connection; and {5} type E - isolated fistula without atresia, also known as H-type tracheoesophageal fistula [13]. In our patient, there were polyhydramnios with type C tracheo-esophageal fistula for which the patient underwent surgical repair.

Renal defects are seen in approximately 50 percent of the patients with VACTERL association. A single umbilical artery is noted in about 35 percent of the patients, which can be associated with urologic abnormalities [6]. Congenital renal anomalies may include renal agenesis, renal dysgenesis, congenital PUJ obstruction, congenital cystic renal disease, horseshoe kidney, cross-fused ectopic kidney, etc [14]. Ultrasonography is the modality of choice for diagnosing fetal renal anomalies [15]. In our patient, there was the presence of a single umbilical artery without the presence of any renal anomaly.

Limb defects are seen in approximately 70 percent of the patients with VACTERL association. They may include polydactyly {extra digits}, syndactyly {fused digits}, or defects of the forearm {absence of bone, absence of digits, radial ray anomaly} [6]. Detailed fetal sonography is paramount for the prenatal detection of limb abnormalities [16]. Differentials for the absence of radius include Holt-Oram syndrome involving the involvement of upper limb abnormalities and cardiac defect; Thrombocytopenia with absent radius (TAR) syndrome; Fanconi anemia characterized by progressive bone marrow failure [17]. Holt-Oram syndrome was ruled out because there was an additional defect of the tracheoesophageal fistula, and the latter two differentials were ruled out after investigating the patient's blood counts, which were within normal limits.

Radial ray anomaly is classified into four subtypes: {1} radial ray type 1- radius is slightly (>2 mm) short, and the hand bends sideways at the wrist (often associated with a hypoplastic thumb); proximal radius usually unaffected; {2} type 2 - the radius bone is very short and the ulna curves sideways and supports the wrist poorly; {3} type 3 - partial absence of radius; and {4} type 4 - complete absence of radius [18]. In our case, we witnessed a type 4 radial ray anomaly with an absent radius and absent thumb with a radial deviation of the hand.

Hydrocephalus or increased CSF accumulation may cause an increase in the pressure of the brain tissues. Symptoms like vomiting, irritability, downward deviation of eyes, and seizures may be noted. Signs of hydrocephalus in newborns include an increase in the head size {macrosomia}. When associated with VACTERL complex association, it is termed as VACTERL-H [19]. Sonography is the first modality of investigation for detecting hydrocephalus because it is a relatively simple and low-risk procedure [20].

In a 10-year follow-up done by Luchtman M et al. [21] on 46 patients with VACTERL association, the estimated mortality rate was 24% and was mainly due to the involvement of the cardiovascular system.

With improvements in the medical and surgical fields, the prognosis of the VACTERL association has improved drastically. However, some patients may have lifetime complications like urinary tract infections, nephrolithiasis in patients with renal anomalies; scoliosis, chronic back pain in patients with vertebral anomalies; fecal incontinence or constipation in patients with anal atresia; gastro-esophageal reflux in patients with tracheo-esophageal fistula.

## Conclusions

VACTERL complex is a rare congenital group of disorders whose distribution is sporadic and non-random. Early detection of the VACTERL complex is vital for the prognosis and survival rate of the patient, and all the components of this complex can be detected antenatally. In the presented case, the patient was initially detected with an absent thumb, radius of the same hand, and tracheo-esophageal fistula. Detection of the involvement of two systems further led to a suspicion of the VACTERL complex association. A thorough check-up showed the presence of ventricular septal defect. Prenatal and postnatal counseling of the parents must be done to explain the complications and prognosis of the defect. Radiological investigation is the primary modality in detecting all the VACTERL defects. The majority of the defects require surgical intervention for an improved prognosis.
